# The Role and Impact of Salivary Zn Levels on Dental Caries

**DOI:** 10.1155/2018/8137915

**Published:** 2018-01-14

**Authors:** Milaim Sejdini, Agim Begzati, Sami Salihu, Sokol Krasniqi, Nora Berisha, Nora Aliu

**Affiliations:** ^1^Faculty of Medicine, Orthodontic Clinic, University of Pristina, Pristina, Kosovo; ^2^Faculty of Medicine, Paediatric Dentistry Clinic, University of Pristina, Pristina, Kosovo; ^3^Faculty of Medicine, Maxillofacial Surgery Clinic, University of Pristina, Pristina, Kosovo

## Abstract

**Introduction:**

Minimal attention has been given to the role of salivary microelements, the importance they have in reducing the intensity of caries, and the effect of caries prophylaxes.

**Aim:**

This research aimed to determine the concentration and quantity of Zn and its impact on the prevention and the reduction of the intensity of caries in schoolchildren aged 12-13 years with permanent dentition.

**Methods:**

For this research, we analyzed the stimulated and nonstimulated full saliva of 106 schoolchildren divided into three groups by mean decayed, missing, and filled teeth (DMFT) index. The control group consisted of 25 caries-free children, the second group had 47 children with mean DMFT index of 1 to 6, and the third group had 34 children with DMFT index of ≥ 6. Complete saliva was collected from all children in a sterile test tube.

**Results:**

The concentration of Zn in saliva before stimulation in caries-free children has variations of the order of 0.001+ to 0.01 mmol/l. The maximum concentration after stimulation is 6.72 mmol/l, while the maximum value is 64.38 mmol/l.

**Conclusion:**

The Zn concentration in the stimulated saliva showed a significant increase in the group of caries-free children and could be described as a positive value for the reduction of caries.

## 1. Introduction

A number of theories attempt to explain the mechanism of initiation of the appearance of caries. Recent research shows that caries have multicausal aetiology, mostly under the influence of general factors but especially under the influence of local factors.

Dental caries is perhaps the most ubiquitous disease that has afflicted mankind. While it is not normally a fatal condition, it can cause a great deal of pain and distress, and the loss of teeth has profound consequences in terms of eating, speaking, and social behavior in general [1]. In recent years, particular importance in the appearance of caries has been devoted to saliva because of the impact of its chemical components and immunology [2, 3].

Although it is known that the basic prerequisites for the appearance of cavities are defined by the presence of microorganisms, the substrate, and the tooth itself for a certain period of time, all of this occurs under the influence of the liquid media of the mouth saliva. In their researches, Gamershtein and Maksimovski [4] and Tvinnereim et al. [5] showed that the appearance of caries is directly affected by the presence of salivary components, specifically the amount of microelements that are present. Saliva, with the presence and composition of its immunochemistry, enables a large number of functions within oral homeostasis such as maintenance of oral cavity humidity and its self-cleaning ability, buffer of oral media, stabilization and preservation of bacterial flora, maintenance and preservation of the surrounding tooth minerals, digestive activity, control of pH, and many other functions.

According to Dawes [6], Mason and Chisholm [7], and Schmidt [8], during high salivary flow, osmolarity can reach plasma osmolarity, while the flow of nonstimulated saliva can be so low that it can reach 1/20 of the plasma osmolarity. Research regarding salivary electrolytes shows that the saliva is saturated with some of the ions. Between the components of salivary electrolytes and those deposited in the enamel of the tooth, it has been found that there are certain equilibrium and controlled report [6–8].

The most important microelements that are present include calcium, sodium, magnesium, zinc, and fluoride; these are of great importance for the mineralization and maturation of hard tooth tissue [9].

The relationship that trace elements in saliva might have with dental caries activity has interested scientists for many years [10].

Qualitative and quantitative analysis (EDS X-rays) shows evidence that the lowest content of the macroelements Ca, P, C, and O and the microelements Al, Cl, In, Mg, Si, Na, S, and W was found in carious enamel layers compared with normal enamel layers [11].

There is an evident difference in salivary electrolyte concentrations from different sources of saliva. Parotid saliva contains fewer Zn electrolytes of Zn and is the opposite of the concentration of Zn^2+^ in the mixed saliva; the concentration of Zn^2+^ varies significantly. The research regarding mineral components in saliva is scarce and has contradictory results with respect to their role in the process of demineralization, remineralization, and dental maturity [12]. In saliva, Zn plays multiple roles and affects many metabolic processes. Its role in the metabolism of protein is so important that it is compared with essential amino acids. It is found in the composition of many enzymes where their activation depends on the presence of Zn. The impact and the amount of Zn in the tooth enamel are more in the outer layer (200–900 ppm) compared to the inner layer (up to 200 ppm) [13–15]. Curzon has noted that zinc and calcium showed promise as antiplaque agents, whereas Sr and Zn may enhance remineralization in enamel [16].

Research has shown that Zn is easily incorporated as a substitute for Ca^++^ ions. Its incorporation in the enamel helps decrease its solubility. In many publications, the role of Zn in dental plaque and oral tissue has been described as an important factor in reducing the ability of bacteria, especially anaerobic bacteria [2, 9].

Zinc salts have antibacterial actions due to their ability to inhibit bacterial adhesion, metabolic activity, and growth [17].

Relatively large amounts of zinc are incorporated into enamel prior to eruption, but after eruption, zinc concentration at the surface of the teeth apparently increases further, suggesting that some incorporation does occur during posteruptive exposure to the oral fluids [18].

Zinc competes with calcium for bacterial-binding sites in model biofilms, and it has been proposed that half of the bound zinc would be released under cariogenic conditions through, for example, protonation of carboxylate and phosphate groups in bacterial lipoteichoic acid [19].

The aim of this research was to find the Zn values in a group of caries-free children and two other groups with vulnerability to caries to determine the concentration and volume of Zn in the full stimulated and nonstimulated saliva through chemical and immunochemical analysis.

Also, this research aimed to define the influence of this microelement in preventing or reducing the rate of the incidence of caries in schoolchildren aged 12-13 years with permanent dentition.

## 2. Materials and Methods

This research was conducted on 106 schoolchildren aged 12 to 13 years with permanent dentition. This was a cross-sectional study where all children were divided into three groups (control group and two groups with vulnerability). The control group was composed of children with all caries-free teeth (DMFT index = 0), healthy oral tissues, and good oral hygiene (25 children). The second group were children with a mean DMFT index of 1 to 6 (47 children), and the third group were children with a mean DMFT index of > 6 (34 children). Nonstimulated saliva was taken from all of the children in the morning because of the circadian rhythm for five-minute duration. For obtaining the stimulated saliva, a clean paraffin wax bone was used for chewing for the duration of five minutes. All samples were taken in sterile test tubes that were graded; until the analysis, the samples were stored in chambers at a temperature of −20°C. Chemical and immunochemical tests were conducted at the Faculty of Science, Ss. Cyril and Methodius University in Skopje. Analyses were performed by flame atomic absorption spectrometer model Solar S4 from Thermo Elemental (UK), at a wavelength of 213.9 nm, spectral slit of 0.5 nm, and lamp current of 10 mA, representing a method with relatively high sensitivity. For the determination of zinc, 1 ml of saliva was diluted with redistilled water in a 10 ml volumetric flask. Statistical analyses were processed with Statistics for Windows/Release 7.0, at the Institute of Statistics of the Faculty of Medicine in Skopje. We obtained permission for this research from the corresponding institutions of our country.

## 3. Results and Discussion

Publications describing the mineral composition of native nonstimulated saliva are few; in the research that has been published, the results are often contradictory. Fluctuations of the volumetric physiological sphere of studied electrolytes are caused by the speed of saliva flow and the composition changes of the various secretions of salivary glands.

Table 1 shows the concentration values of the examined samples and the amount of Zn in saliva before and after stimulation with paraffin. The first group (control) had a concentration of Zn in stimulated saliva that varied in the interval of 0.01 ± 0.01 mmol/l, with a confidence interval of −0.01 + 0.01, a minimum value of 0.0002, and a maximum value of 0.03 mmol/l. The Zn concentration in the stimulated saliva showed variations in the interval of 0.28 ± 1.35, with a confidence interval of −0.27 + 0.83 mmol/l. The minimum value was 0.002, while the maximum value was 6.78 mmol/l. The Zn amount before stimulation was 0.02 ± 0.02 *µ*mol/l. The minimum value is 0.0001 *µ*mol/l, while the maximum value is 0.07 *µ*mol/l. The amount of Zn after stimulation showed a more pronounced change with values of 2.65 *µ*mol/l, with a standard deviation of ± 12.86, a confidence interval of −2.66 + 7.96, and a maximum value of 64.38 *µ*mol/l.

Table 2 shows the differences in the concentration and quantity of Zn before and after stimulation. The concentration of Zn after stimulation was *Z* = 0.55 and showed no increase compared with that prior to stimulation (*p* < 0.05), while the amount of Zn was *Z* = 3.56 and showed a significant increase after salivary stimulation (*p* < 0.001).

A reported decrease in the molarities of Ca and Zn that are found in caries can be very important in acknowledging the risk of caries, namely, to have an important role in the demineralization process of tooth decay. A relative surplus of Zn over Ca can be more closely associated with the occurrence of caries because it is validated with statistical tests.

Table 3 shows the values of Zn concentration and the quantity before and after stimulation in children with DMFT index of 1–6. The Zn concentration varies before stimulation at intervals of 0.01 ± 0.01 mmol/l, with a confidence interval of −0009 + 0.01, a minimum value of 0.0002, and a maximum value of 0.08 mmol/l. The Zn concentration after stimulation has variations with intervals of 0.01 ± 0.01 mmol/l, with a confidence interval of −0005 + 0.01, a minimum value of 0.0005, and a maximum value of 0.07 mmol/l. The Zn amount before the stimulation varied at intervals of 0.02 ±0.03 *µ*mol/l, with a confidence interval of −0.01 + 0.03, a minimum value of 0.0005, and a maximum value of 0.16 *µ*mol/l. The amount of Zn after stimulation varies, ranging between 0.07 ± 0.12 *µ*mol/l, with a confidence interval of −0.03 + 0.11, a minimum value of 0.002, and a maximum value of 0.71 *µ*mol/l.

The highest incidence of enamel lesions was observed in the mandibular molars in rats fed with Zn-deficit diets compared with mice doubly fed with supplementary Zn diets. Furthermore, the effects of Zn deficiencies in caries in young mice were observed in a greater mass in the smooth surface of molar zinc. Dietary Zn may be a trace mineral that is important during the posteruptive process of enamel mineralization; it could reduce a tooth's sensitivity to caries.

Table 4 shows the differences in Zn concentration and quantity before and after saliva stimulation. For *Z* = 1.84, no significant changes were observed in the Zn concentration after stimulation (*p* < 0.05). The amount of Zn for *Z* = 3.36 is significantly higher after stimulation with high valuation (*p* < 0.001).

Table 5 shows the values of Zn concentration and quantity before and after stimulation of the third group of researched children with a mean DMFT index of > 6.

The Zn concentration before and after stimulation in the saliva did not show significant differences; the amount of Zn before and after stimulation showed significance in the saliva after stimulation for the interval of 0.04 ± 12.08 *µ*mol/l, with a confidence interval of −0.01 + 0.07, a minimum value of 0.0006, and a maximum value of 12.48 *µ*mol/l.

Differences between Zn concentration and quantity before and after stimulation are presented in Table 6. The concentration of Zn after stimulation for *Z* = 0.84 did not show important differences (*p* < 0.05); the amount of Zn for *Z* = 3.65 significantly increased, showing statistical significance (*p* < 0.001).

Differences were observed among the three groups in this research (Table 7) for *H* = 2.54 (*p* < 0.05); for *H* = 3.56, no significant difference was found in the Zn concentration before and after saliva stimulation nor in the nonstimulated saliva (for *H* = 5.66 and *p* < 0.05). A difference was observed among the three groups in the Zn amount after stimulation for *H* = 7.99 (*p* < 0.05).

The amount of Zn for *U* = 568.00 (Table 8) among the groups before stimulation is obviously higher in the second group compared to the third group (*p* < 0.05), while for *U* = 240, the amount of Zn after stimulation has had an ascendance of great importance in the first group compared to the third group (*p* < 0.01).

Although we expected that there will be differences in the three groups for *H* = 5.66, we did not find a statistical significance during the comparison. Differences for *U* = 568 and *U* = 240 in the three groups showed a statistical significance at *p* < 0.01 and *p* < 0.05, respectively.

During the research, we saw that Zn was easily incorporated in the hydroxyapatite, exchanging with the Ca^+2^ ions. With the incorporation of Zn, the dissolution of enamel decreases, but this does not affect the appearance of caries [20]. Insufficient data to describe the role of Zn in the process of caries have been presented in the literature. Quantitative analysis of ions released into solution following the demineralization of samples confirmed that Zn reduces the rate of demineralization as a function of concentration. To influence enamel demineralization under cariogenic conditions, Zn must be available in the plaque fluid at a concentration sufficient to reduce or inhibit tooth mineral loss [21]. Contrary to the research done by Mohammed et al. [21], a study by Duggal et al. found that the concentration of Zn had no relationship with dental caries [22].

The results of Bales and Freeland-Graves [23], Fang et al. [14], and Gregory et al. [24] showed that, in the mice fed with a diet deficient in Zn, the incidence of caries has been high in the molars compared to the mice fed with normal food. This shows a close connection of Zn with proteins. Although there is little research on the role of Zn in the process of decay, in our research, the concentration of Zn is significantly higher in the saliva of children with caries where quantitative growth of Zn is evident in the two groups of children; this could, together with the “empty spaces,” have the potential to show the demineralization role of saliva. It is assumed that Zn is easily released from the crystalline structure by leaving “empty space.” Our results are similar to those of Tvinnerein et al. [25]. Regarding the clinical effects of zinc on de- and remineralization, it seems unlikely that potentially beneficial effects, such as reductions in solubility and enhanced/prolonged lesion porosity to mineral ingress, will counter any possible negative effects [26]. In light of the current findings, it would appear that there is scope for exploring and optimizing the therapeutic potential of zinc, not only as an antibacterial agent but also as a possible preventive treatment for caries [21]. Distinguished from the results of other authors [27, 28], our data do not match since they have worked in selective saliva (from the saliva of the selected gland, with incomplete saliva) and because of the indirect influence of saliva on dental plaque. Differences were also prescribed to children's age and the presence of mixed dentition. According to Hussein et al., salivary Cu and Zn levels were significantly higher in children with dental caries compared to those who were caries-free [29]. Also, it is found that the use of toothpaste containing nanocrystals of carbonate hydroxyapatite replaced with Zn can produce mimicking effect of morphology, structure, and composition of biological hydroxyapatite of enamel [30].

## 4. Conclusion

Based on the results obtained with the chemical and immunochemical analysis of whole saliva, we can obtain the following conclusions:

- After the stimulation, we found that the Zn concentration in the first group was higher.
- The quantity of Zn before and after the stimulation in the second and third groups with caries showed statistically significant differences.
- The quantity of Zn after the stimulation showed significant differences among the three groups. These differences are higher in the first group in comparison to the second and third groups.
- The increase in Zn concentration and quantity in the first group (caries-free) in comparison with the second and third groups indicates the positive effect on reducing caries.

From these findings, we can conclude that Zn has an impact in reducing the appearance of caries that is proved with the Zn quantity differences found in the three groups that were investigated.

## Figures and Tables

**Table 1:** Concentration and quantity of Zn before and after stimulation in caries-free children.

Parameters	*N*	Mean	Confidence −95.00%	Confidence 95.00%	Minimum	Maximum	SD
Zn concentration, mmol/l (before the stimulation)	25	0.01	−0.01	0.01	0.0002	0.03	0.01
Zn concentration, mmol/l (after the stimulation)	25	0.28	−0.27	0.83	0.002	6.78	1.35
Zn quantity (*µ*mol/l): before the stimulation	25	0.02	−0.01	0.03	0.0001	0.07	0.02
Zn quantity (*µ*mol/l): after the stimulation	25	2.65	−2.66	7.96	0.02	64.38	12.86

**Table 2:** Zn concentration before and after the stimulation.

Parameters	*N*	*T*	*Z*	Found *p*	*p*	Sig./N. sig.
Zn concentration (before/after stimulation)	25	142	0.55	0.58	>0.05	N. sig.
Zn quantity (before/after stimulation)	25	30	3.56	0.0003	<0.001	Sig.

Sig = significant, N. sig = not significant.

**Table 3:** Concentration and quantity of Zn before and after stimulation in children with DMFT index of 1–6.

Parameters	*N*	Mean	Confidence −95.00%	Confidence 95.00%	Minimum	Maximum	SD
Zn concentration, mmol/l (before the stimulation)	47	0.01	0.009	0.01	0.0002	0.08	0.01
Zn concentration, mmol/l (after the stimulation)	47	0.01	0.005	0.01	0.0005	0.07	0.01
Zn quantity (*µ*mol/l): before the stimulation	47	0.02	0.01	0.03	0.0005	0.16	0.03
Zn quantity (*µ*mol/l): after the stimulation	47	0.07	0.03	0.11	0.002	0.71	0.12

**Table 4:** Zn concentration before and after the stimulation in children with DMFT index of 1–6.

Parameters	*N*	*T*	*Z*	Found *p*	*p*	Sig./N. sig.
Zn concentration (before/after stimulation)	47	390	1.84	0.065	>0.05	N. sig.
Zn quantity (before/after stimulation)	47	246	3.36	0.0007	<0.001	Sig.

**Table 5:** Concentration and quantity of Zn before and after stimulation with mean DMFT index of  >6.

Parameters	*N*	Mean	Confidence −95.00%	Confidence 95.00%	Minimum	Maximum	SD
Zn concentration, mmol/l (before the stimulation)	34	0.008	0.005	0.01	0.0002	0.03	0.008
Zn concentration, mmol/l (after the stimulation)	34	0.007	0.003	0.01	0.0002	0.05	0.009
Zn quantity, *µ*mol/l (before the stimulation)	34	0.01	0.007	0.02	0.0005	0.07	0.01
Zn quantity, *µ*mol/l (after the stimulation)	34	0.04	0.01	0.07	0.0006	0.49	0.08

**Table 6:** Zn concentration before and after stimulation in children with mean DMFT index of >6.

Parameters	*N*	*T*	*Z*	Found *p*	*p*	Sig./N. sig.
Zn concentration (before/after stimulation)	34	248	0.84	0.39	>0.05	N. sig.
Zn quantity (before/after stimulation)	34	84	3.65	0.0002	<0.001	Sig.

**Table 7:** Statistical significance for the analyzed parameters between 3 groups.

Parameters	DMFT index	*H*	Found *p*	*p*	Sig./N. sig.
Zn concentration, mmol/l (before stimulation)	<1	2.54	0.28	>0.05	N. sig.
	1–6				
	>6				
Zn concentration, mmol/l (after stimulation)	<1	3.56	0.16	>0.05	N. sig.
	1–6				
	>6				
Zn quantity, *µ*mol/l (before stimulation)	<1	5.66	0.05	>0.05	N. sig.
	1–6				
	>6				
Zn quantity, *µ*mol/l (after stimulation)	<1	7.99	0.01	<0.05	Sig.

**Table 8:** Significant differences among analyzed groups.

Parameters	Compartments between groups	*U*	Found *p*	*p*	Sig/N. sig
Zn quantity, *µ*mol/l (before the stimulation)	Gr_2_/Gr_3_	568.0	0.02	<0.05	Sig.
Zn quantity, *µ*mol/l (after the stimulation)	Gr_1_/Gr_3_	240.0	0.004	<0.01	Sig.
